# THROUGH THE LOOKING-GLASS: THE FAMILY SHAME OF ELDER ABUSE

**DOI:** 10.1093/geroni/igad104.2130

**Published:** 2023-12-21

**Authors:** Jessica Hsieh, Raza Mirza

**Affiliations:** University of Toronto, Toronto, Ontario, Canada; University of Toronto, Toronto, Ontario, Canada

## Abstract

Elder abuse (EA) has been recognized as a serious public health concern. Recent studies have found that approximately 10% of community-dwelling, cognitively intact older adults experience some form of EA each year. Although EA research has made substantial progress, EA is often under-reported, with only an estimated 15% of cases being reported to formal support services. One of the main reasons for the under-reporting of EA is the victims’ feelings of shame, which have been shown to be exacerbated when the perpetrator is a family member and/or an individual with whom the older adults have close trusting relationships. Qualitative interviews conducted with 12 older adult (parent) and adult child caregiver dyads (n=24) revealed that older adults who experience EA by their adult children experience intense shame. Thematic analysis focused on what led to these feelings of shame, and this resulted in four main themes: (1) Failure in their role as a parent; (2) Adult children viewing them as powerless and unworthy; (3) Experiencing negative psychological effects; and (4) Self-blame. With a sense of responsibility to protect their family, older adults tend to keep ‘family shame’ to themselves, leading to a reluctance to disclose EA. As spouses and adult children, the two most common caregiving types, have also been reported to be the two most common groups of EA perpetrators, gaining a better understanding of the root causes of shame can help to support older adults in living safely in their homes and communities for as long as possible.

